# A duplex tetra-primer ARMS-PCR assay to discriminate three species of the *Schistosoma haematobium* group: *Schistosoma curassoni, S. bovis, S. haematobium* and their hybrids

**DOI:** 10.1186/s13071-023-05754-9

**Published:** 2023-04-07

**Authors:** Manon Blin, Sarah Dametto, Privat Agniwo, Bonnie L. Webster, Etienne Angora, Abdoulaye Dabo, Jérôme Boissier

**Affiliations:** 1 Hosts Pathogens Environment Interactions, UMR 5244, CNRS, IFREMER, UM, University of Perpignan Via Domitia, Perpignan, 66860 France; 2 SAS ParaDev®, 66860 Perpignan, France; 3 Department of Epidemiology of Infectious Diseases, Faculty of Pharmacy, IRL 3189, University of Sciences, Techniques and Technologies of Bamako, Bamako, Mali; 4 Wolfson Wellcome Biomedical Laboratories, Department of Science, Natural History Museum, London, SW7 5BD UK; 5 London Centre for Neglected Tropical Disease Research, Imperial College London School of Public Health, London, W2 1PG UK; 6 Swiss Tropical and Public Health Institute, P.O. Box, 4002 Basel, Switzerland; 7 University of Basel, Kreuzstrasse 2, 4123 Allschwil, Switzerland; 8 Unité de Formation et de Recherche Sciences Pharmaceutiques et Biologiques, Université Félix Houphouët-Boigny, BPV 34 Abidjan, Côte d’Ivoire

**Keywords:** T-ARMS-PCR, Schistosomiasis, SNPs, Genotyping, Hybridization

## Abstract

**Background:**

The use of applications involving single nucleotide polymorphisms (SNPs) has greatly increased since the beginning of the 2000s, with the number of associated techniques expanding rapidly in the field of molecular research. Tetra-primer amplification refractory mutation system—PCR (T-ARMS-PCR) is one such technique involving SNP genotyping. It has the advantage of amplifying multiple alleles in a single reaction with the inclusion of an internal molecular control. We report here the development of a rapid, reliable and cost-effective duplex T-ARMS-PCR assay to distinguish between three *Schistosoma* species, namely *Schistosoma haematobium* (human parasite), *Schistosoma bovis* and *Schistosoma curassoni* (animal parasites), and their hybrids. This technique will facilitate studies of population genetics and the evolution of introgression events.

**Methods:**

During the development of the technique we focused on one of the five inter-species internal transcribed spacer (ITS) SNPs and one of the inter-species 18S SNPs which, when combined, discriminate between all three *Schistosoma* species and their hybrid forms. We designed T-ARMS-PCR primers to amplify amplicons of specific lengths for each species, which in turn can then be visualized on an electrophoresis gel. This was further tested using laboratory and field-collected adult worms and field-collected larval stages (miracidia) from Spain, Egypt, Mali, Senegal and Ivory Coast. The combined duplex T-ARMS-PCR and ITS + 18S primer set was then used to differentiate the three species in a single reaction.

**Results:**

The T-ARMS-PCR assay was able to detect DNA from both species being analysed at the maximum and minimum levels in the DNA ratios (95/5) tested. The duplex T-ARMS-PCR assay was also able to detect all hybrids tested and was validated by sequencing the ITS and the 18S amplicons of 148 of the field samples included in the study.

**Conclusions:**

The duplex tetra-primer ARMS-PCR assay described here can be applied to differentiate between *Schistosoma* species and their hybrid forms that infect humans and animals, thereby providing a method to investigate the epidemiology of these species in endemic areas. The addition of several markers in a single reaction saves considerable time and is of long-standing interest for investigating genetic populations.

**Graphical Abstract:**

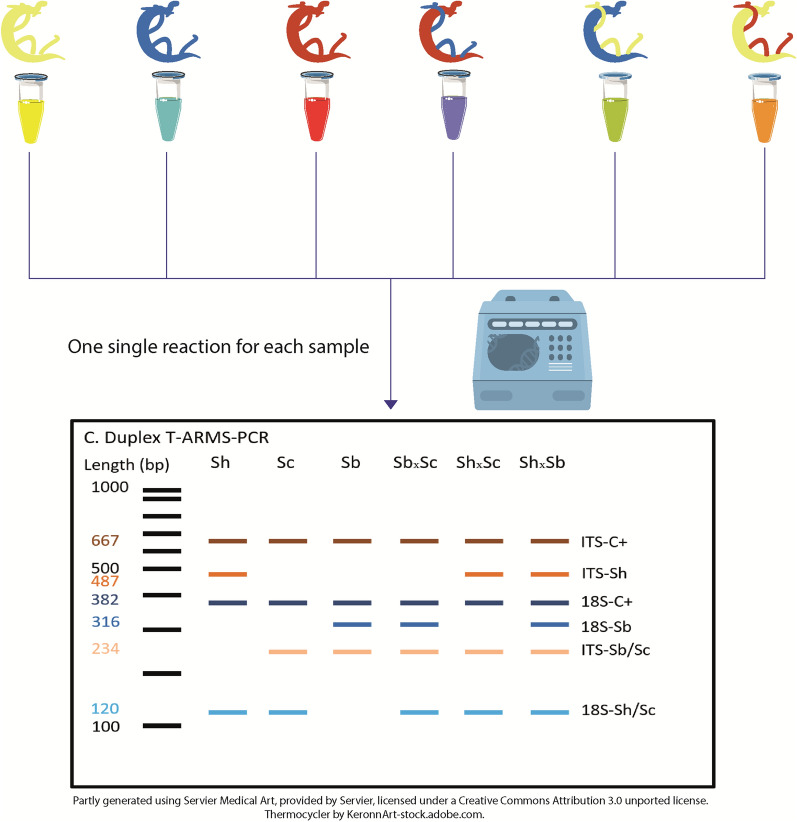

## Background

Since the 1980s, the use of molecular markers and associated analytical methods has been steadily increasing, not only within the research environment but also in fields such as human and environmental diagnostics. Among these techniques, single nucleotide polymorphisms (SNPs) applications have increased dramatically since the beginning of the 2000s, allowing expansion in various research fields [1]. SNPs are defined as a single-base DNA substitution in an individual’s genome which, by definition, are found in > 1% of the population [2]. Intra- or inter-specific SNPs are the most common type of genetic variation between individuals, with a frequency of approximately every 800 base pairs throughout the genome [3]. The identification of SNPs is widely used in several domains, ranging from medicine to agriculture. SNP genotyping is one of the keys methods underlying a number of medical diagnoses as many SNPs are linked to diseases, such as cancer, or involved in drug resistance [4, 5]. Indeed, SNPs sometimes occur in gene coding regions where they are bi-allelic and can cause missense or nonsense mutations that can result in several diseases [6]. They are also employed to probe the functional consequences of missense and noncoding changes made by CRISPR/Cas9 gene editing research [7]. SNPs occur more frequently in noncoding regions but can have an equally phenotypic impact if they are in regulatory regions [8]. Also, SNPs have been the markers of choice for > 20 years in taxonomy, systematics, phylogenetics and historical demography studies [9, 10]. The development of diagnostic molecular markers has long been of interest to population geneticists as a means of clarifying taxonomic uncertainties [11]. Hybridization and introgression events between species are common evolutionary phenomena [12], and SNPs are the simplest way to estimate the ancestry of potentially hybridized individuals by using taxon-specific diagnostic alleles [13].

SNP genotyping methods will vary depending on the type of analysis being performed. The evolutionary analyses of many species usually involves analyses of SNPs on a genome-wide scale using next-generation sequencing (NGS) methodologies [14]; for example, 2.5 million SNPs have been determined by whole-genome sequencing in *Drosophila melanogaster*, a model species [15]. However, NGS technologies are also used in studies in non-model species, such as trematodes, where SNPs allow species or population discrimination [16]. The methods for high-throughput genotyping are now numerous and include, for example, Taqman technology [17], microarrays [18] and MALDI-TOF mass spectrometry [19]. However, the determination of all SNPs in the genome is not actually necessary for species identification. The difficulty lies in the prerequisite for SNP genotyping, namely the identification of taxon-specific diagnostic alleles (SNPs with sufficient interspecific variability but intraspecifically conserved). Several methods have been developed to genotype taxon-specific diagnostic SNPs, with most of these using DNA amplification followed by an analysis method, such as restriction fragment length polymorphism analysis of PCR amplified fragments (PCR–RFLP). This method works on the principle of enzymatic cleavage of the amplification products common to both alleles, which makes it possible to obtain fragments of a specific size for each allele [20]. Therefore, the process requires the time needed for a standard PCR followed by one or more enzymatic digestions. High-resolution melting (HRM) analysis is another PCR-based technique capable of determining SNPs. This method depends on the melting temperature of DNA amplicons in the presence of saturating DNA-binding dyes [21, 22]. However, because this method requires a real-time fluorescence reader and fluorescent probes, it can be expensive and time-consuming. LOOP-mediated isothermal amplification (LAMP) has been demonstrated to be a simpler, cheaper and faster alternative to the classical PCR-based method for SNP identification [23]. The advantage of this method is that the internal primers of the reaction at positioned at the polymorphism and, consequently, amplification takes place only on the specific allele [24, 25]. The major drawback is that there is no amplification control since the result for the non-specific, or non-mutant, allele is negative [24, 25]; therefore, a second and complementary reaction must then be performed to ensure the efficiency of the technique, or the two alleles must be multiplexed by including probes [26].

**Fig. 1:**
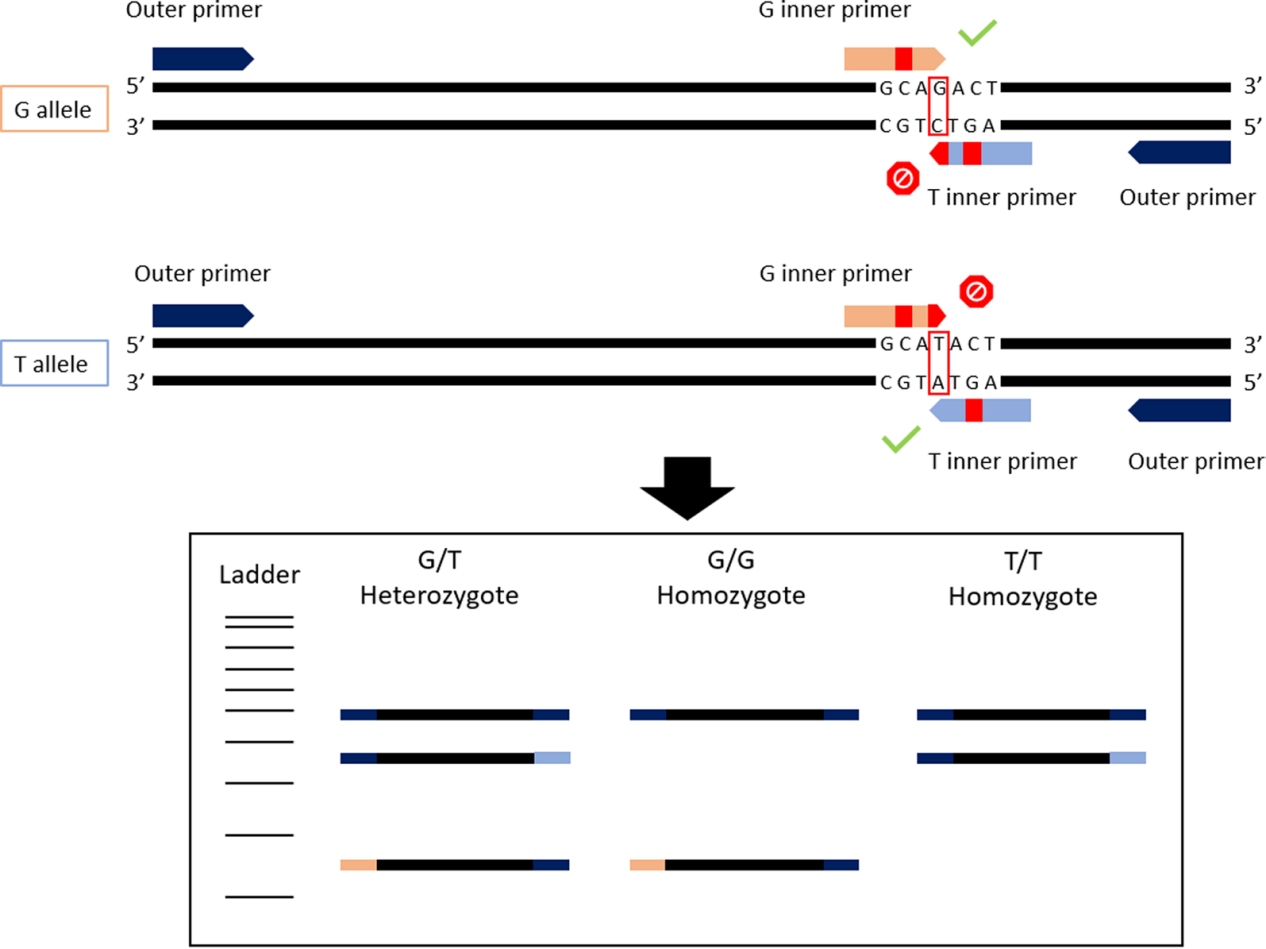
Schematic illustration of the T-ARMS-PCR assay for single nucleotide polymorphism (SNP) genotyping and virtual gel patterns. The targeted SNP is highlighted in red, as are the mismatches with the primers’ sequences. T–ARMS–PCR, Tetra-amplification refractory mutation system PCR

Another SNP genotyping method is tetra-primer amplification refractory mutation system—PCR (T-ARMS-PCR); this method for genotyping SNPs combines the advantages of amplifying both alleles in one reaction and of including an internal reaction control. T-ARMS-PCR has been developed by combining certain principles of the tetra-primer PCR [27] and the ARMS-PCR [28]. This technique consists of an amplification of three fragments of different lengths by four primers: (i) a common outer primer pair that amplifies the entire target from both alleles; and (ii) two inner primers overlapping on their 3’ extremity, located at the SNP, with each primer being specific to one of the two alleles (Fig. 1). A mismatch is voluntarily added at position-3 from the 3’ terminus of the inner primers to destabilize the base pairing between the primers and their non-targeted allele [29]. Then, each specific internal primer with each external primer produces a amplicon of allele-specific size by placing the outer primers at different distances from the SNP [30]. Agarose gel electrophoresis is then used to distinguish homozygotes that present two fragments, the common control and the allele-specific fragment of a different size, from heterozygotes which will present three fragments (Fig. 1) [29].

Schistosomiasis is a neglected tropical disease that affects 230 million people worldwide. This disease is caused by trematodes of the genus *Schistosoma* which require a mammalian definitive host and gastropod as intermediate host. Six *Schistosoma* species are of medical importance (*S. mansoni, S. haematobium, S. intercalatum, S. guineensis, S. japonicum* and *S. mekongi*) while a number of other species, such *S. bovis*, *S. mattheei* or *S. curassoni*, are of veterinary importance. A growing number of field studies have provided evidence for hybridization events between sympatric *Schistosoma* species in Africa [16, 31–36], with hybridization more frequently found between species within the same *Schistosoma* clade (closely related species) than between clades (distantly related species). Most of the *Schistosoma* hybrids have been identified in the *S. haematobium* clade that includes nine closely related species. Hybrids have also been found between *S. haematobium* and *S. bovis*; *S. haematobium* and *S. curassoni*; *S. haematobium* and *S. guineensis*; *S. curassoni* and *S. bovis*; and *S. haematobium* and *S. mattheei* [31, 32, 34, 37–39]. Several studies suggest the possibility that interspecific crosses can generate lineages with increased virulence compared to parental species (i.e. hybrid vigour) and lead to increased parasite fitness, such a broader host spectrum [16, 32–35, 40, 41]. This has been evidenced in *S. bovis* × *S. haematobium* parasite crosses infecting humans, livestock, and rodents in Benin [42]. Hybridization between parasites can be the cause of the emergence and rapid evolution of new zoonotic diseases and is an important challenge for the prevention, control and therapeutic treatment of these diseases [37, 41, 43, 44]. The possible consequences of hybridization have highlighted a need to develop a new, faster, simpler and more efficient diagnostic for hybrids [16]. Hybrids and species can only be accurately identified using molecular methods which involve the analyses of both nuclear and mitochondrial molecular markers. The markers currently used to genotype *Schistosoma* hybrids are the cytochrome oxidase subunit 1 mitochondrial gene (*cox*1) and the nuclear markers internal transcribed spacer (ITS) and 18S [32, 34, 38, 39]. Discordance in species assignation between the mitochondrial and nuclear markers, and heterozygous profiles for the nuclear markers enables discrimination of hybrids/introgressed forms of *Schistosoma*. For the mitochondrial *cox*1 marker, species identification is often performed using the rapid diagnostic (RD)-PCR described by Webster et al. [45], but this method cannot discriminate *S. bovis* from *S. curassoni* [46]. Similarly, the ITS PCR-RFLP cannot discriminate *S. bovis* and *S. curassoni* [30, 32, 37, 38]*,* with Sanger sequencing needed for full confirmation of identity. Moreover, other nuclear regions, namely the 18S, is needed for further species/hybrid identification [47]. The aim of this study was to develop a single duplex T-ARMS-PCR assay, targeting two DNA regions, the ITS and the 18S, to discriminate between *S. haematobium*,* S. bovis*,* S. curassoni* and their hybrid forms.

## Methods

### Biological material

Table 1 lists the biological material included in this study. We used either worms or miracidia from three distinct species known to hybridize: *S. curassoni*, *S. bovis* and *S. haematobium*. These three *Schistosoma* species are clustered in the same monophyletic *S. haematobium* group, and all hybrid combinations have been molecularly detected in the field [38, 39, 48]. The biological material included laboratory-maintained or field-collected samples. Of the 160 field-collected *Schistosoma* samples, 148 samples, regardless of life-cycle stage (miracidium or adult), were genetically characterized by Sanger sequencing of the nuclear ITS and 18S ribosomal DNA regions and of a partial region of the mitochondrial *cox*1 gene, to confirm their species or hybrid form (Table 1).

**Table 1:** Biological samples used in the study

Genotype^a^				Origin			
ITS	18S	*cox*1 mitotype	Genotype^a^	Laboratory/field isolate	Country	Life-cycle stage	*n*
Sh	Sh	Sh	Sh	Laboratory	Egypt	Adult	3
Sh	Sh	Sh	Sh	Field	Mali^c^	Miracidia	58
Sb	Sb	Sb	Sb	Laboratory	Spain	Adult	3
Sb	Sb	Sb	Sb	Field	Mali	Adult	37
Sc	Sc	Sc	Sc	Field	Mali	Adult	31
Sc	Sc	Sc	Sc	Field	Senegal	Adult	12
Sh and (Sb or Sc^b^)	Sh and Sb	Sb	Hybrid Sh/Sb	Laboratory F1	Egypt/Spain	Adult	3
Sh and (Sc or Sb^b^)	Sh and Sc	Sh	Hybrid Sh/Sc	Field	Mali^c^	Miracidia	19
Sh and (Sc or Sb^b^)	Sh and Sc	Sh	Hybrid Sh/Sc	Field	Ivory Coast^d^	Miracidia	3

*cox1*, Cytochrome oxidase subunit 1 mitochondrial gene,* ITS* internal transcribed spacer,* n* number of individuals,* Sb* *Schistosoma bovis*,* Sc* *Schistosoma curassoni*,* Sh Schistosoma haematobium*,* SNP* single nucleotide polymorphism, * T–ARMS* tetra-primer amplification refractory mutation system

^a^The genetic profiles are inferred by Sanger sequencing of the T-ARMS regions of the ITS, 18S and by the rapid diagnostic multiplex PCR (RD-PCR) region of the *cox*1 DNA [45]

^b^The sequence of the ITS or 18S cannot be distinguished between the two species as the region analysed does not contain species-specific SNPs

^c^Ethical clearance was obtained from the Faculty of Medicine, Pharmacology, and Odonto-Stomatology of Mali (reference no.2018/71/CE/FMPOS)

^d^Ethical clearance was obtained from the Ministry of Health and Public Hygiene in Ivory Coast (reference no.003–18/MSHP/CNER-kp) [38]

### DNA extraction

DNA was extracted from individual adult worms using the E.Z.N.A® Tissue DNA Kit (Omega Bio-Tek Inc., Norcross, GA, USA) according to the manufacturer’s instructions. DNA was eluted from each sample in 50 µl of water at 50 °C. The DNA concentration of each sample was measured using a Qubit® 2.0 fluorometer (Thermo Fisher Scientific, Waltham, MA, USA). The DNA for each sample was normalized to 0.5 ng/µl using ultrapure water.

Miracidia were first stored on FTA cards and then DNA was extracted according to a modified version of a previously published protocol [49]. Briefly, each 2-mm disc, cut from FTA card, was suspended in 50 µl of ultrapure water at room temperature for 10 min. The water was then discarded, and 80 µl of 5% Chelex® solution was added to the samples which were then incubated for 30 min at 65 °C with agitation, followed by incubation at 99 °C for 8 min without agitation. The samples were then centrifuged at 14,000 rpm for 2 min, and 50 µl of supernatant containing the DNA was recovered from each sample. The resulting DNA extracts and dilutions were stored at − 20 °C for subsequent molecular analyses.

### Primer design

Two T-ARMS-PCR assays targeting two distinct nuclear DNA regions, ITS and 18S, were designed. The ITS and 18S regions contain species-specific SNPs that can be used to discriminate the three *Schistosoma* species being investigated [47] (Table 2).

**Table 2:** The internal transcribed sequence and 18S single nucleotide polymorphisms discriminating the three *Schistosoma* species and the hybrid forms

SNP and *Schistosoma* species	DNA region									
	ITS					18S				
SNP position	51	*703*	758	808	878	225	250	*297*	685	1184
*S. haematobi*um	A	*G*	C	G	C	T	C	*T*	T	A
*S. bovis*	G	*A*	T	A	T	C	T	*C*	C	G
*S. curassoni*	A	*A*	T	A	T	T	T	*T*	T	G

Hybrids can be observed to have alleles from both species in the combination, but this depends on the hybrid generation and the DNA marker being used

The SNPs targeted by the ITS and 18S T-ARMS-PCRs are shown in italics

ITS and 18S sequence data available from Genbank (ITS accession numbers GU257398.1, MT580950.1 and MT580946.1; 18S accession numbers Z11976.1, AY157238.1 and AY157236.1 for *S. haematobium*, *S. bovis* and *S. curassoni*, respectively) were used for the primer design, which was carried out using the programme developed by Ye [27] (available at: http://primer1.soton.ac.uk/primer1.html) following the authors’ recommendations. The primers were designed taking the following constraints into account: (i) they should be located far from the DNA sequence extremities; (ii) the targeted SNP should be preferentially C/T or G/A [30]; (iii) the combination of two targeted SNPs have to discriminate all three species and their hybrids (see Table 2); (iv) the minimum number of bases between two targets, within or between the two T-ARMS-PCRs, should be 50 bp; and (v) the amplified fragment sizes for each primer set need to differ by at least 50 bp in length (e.g. 100–400 bp for the 18S and 200–700 bp for the ITS) so that they can be easily separated and identified by agarose gel electrophoresis. Geneious Prime® version 2022.0.2 (Biomatters, Auckland, New Zealand) was used to make adjustments to the primer sets to obtain sufficiently different fragment sizes between the two genes. The primer set outputs for the ITS and 18S target genes, from the programme designed by Ye [27], were entered into Geneious Prime for in silico T-ARMS-PCR design, with replacement outer primers generated to obtain the desired amplicon sizes. Estimation of the primer dimer and stability of each primer set were checked using the "Multiple Primer Analyzer" (Thermo Scientific Web Tool; Thermo Fisher Scientific) and the OligoAnalyzer™ Tool (IDT™; Integrated DNA Technologies, Inc., Coralville, IA, USA). All of the primers selected were synthesized by Sigma-Aldrich, Merck (Darmstadt, Germany). The designed sets of primers are presented in Table 3. The ITS T-ARMS-PCR primer set enables discrimination of *S. bovis* and *S. curassoni* (amplicon size 234 bp) from *S. haematobium* (amplicon size 487 bp), and the 18S T-ARMS-PCR primer set enables discrimination of *S. curassoni* and *S. haematobium* (amplicon size 120 bp) from *S. bovis* (amplicon size 316 bp). Each primer set was designed so that the non-species-specific amplicon was always amplified and visualized, acting as an internal control and preventing false negative identifications.

**Table 3:** Tetra-primer amplification refractory mutation system-PCR primer sets for the identification of *S. haematobium*, *S. bovis* and *S. curassoni* and their hybrids

DNA target	T-ARMS-PCR primer set^a^	Sequence	Working concentration (µM)	Melting temperature (Tm; °C)	Length (bp)
*ITS*					
Outer	OR-ITS	TCGTGCGTATTACACACACCATCGGTACAAACC	12	64	33
	OF-ITS	GCATGCAAATCCGCCCCGTTATTGTTCCT	12	64.7	29
Inner	IR-Sh-ITS	TGTGGCCGGACTATTAGGACGGAGCCGTC	15	67.5	29
	IF-Sb/Sc-ITS	CGCATATCAACGCGGGTTGCTGGGCA	15	67.3	26
*18S*					
Outer	OR-18S	GCACCAGACTTGCCCTCCAATTGGTCC	12	65.2	27
	OF-18S	GCATTTATTAGAACAGAACCAAYCGGGCG	12	59.8/61.5	29
Inner	IR-Sc/Sh-18S	ATTTGAAAGATCCGTCGCCGACAAAA	15	59.3	26
	IF-Sb-18S	TGGATAACTTTACTGATCGCAGTCGGACC	15	61.2	29

OF and OR define forward and reverse external primers, respectively. IF and IR define internal forward and reverse primers, respectively. The outer primers for each target generate the internal control *Schistosoma* sp. amplicon, whereas the inner primer together with the outer primers generate the species-specific amplicons

^a^IR-Sh-ITS + OF-ITS generates the ITS *S. haematobium* amplicon. IF-Sb/Sc-ITS + OR-ITS generates the ITS *S. bovis or S. curassoni* amplicon. IR-Sc/Sh-18S + OF-18S generates the 18S *S. haematobium* or *S. curassoni* amplicon. IF-Sb-18S + OR-18S generates the 18S *S. bovis* amplicon

**Fig. 2:**
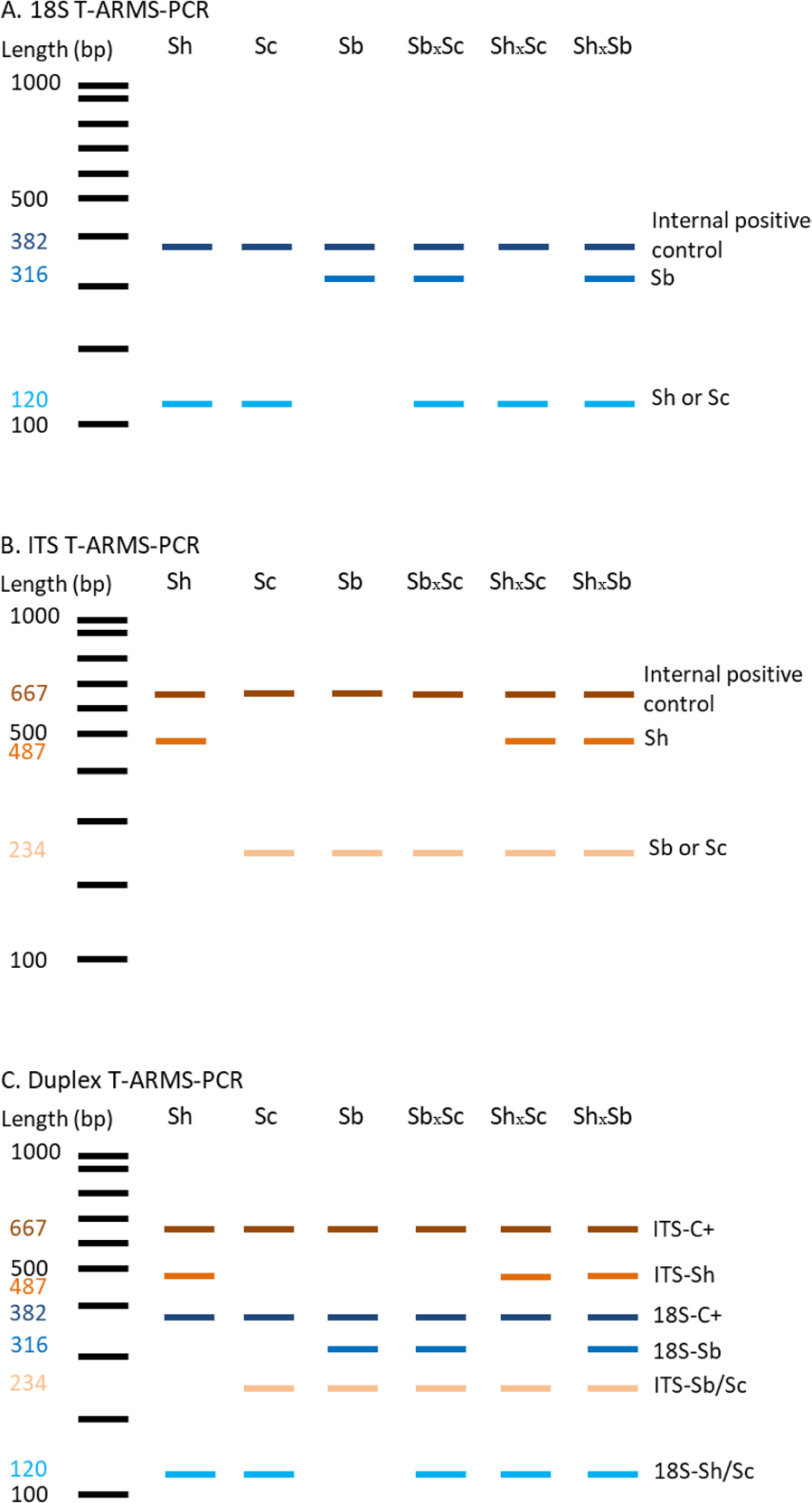
Virtual gels of 18S T-ARMS-PCR (**a**) internal transcribed sequence (*ITS*) T-ARMS-PCR (**b**) and duplex T-ARMS-PCR (**c**). The 382- and 667-bp amplicons are the internal positive controls for the *Schistosoma* sp. 18S and ITS DNA regions, respectively. The 18S T-ARMS-PCR was designed to distinguish *Schistosoma bovis* (*Sb*) from *Schistosoma curassoni* (*Sc*) or* Schistosoma haemobium* (*Sh*), whereas the ITS T-ARMS-PCR was designed to distinguish Sh from Sb or Sc. When combined in duplex, the ITS/18S T-ARMS-PCR should provide unique amplicon profiles for each species (Sh: 120, 382, 487 and 667 bp; Sc: 120, 234, 382 and 667 bp; Sb: 234, 316, 382, and 667 bp) and/or hybrid forms (Sb×Sc: 120, 234, 316, 382 and 667 bp; Sh×Sc: 120, 234, 382, 487 and 667 bp; and Sh×Sb: 120, 234, 316, 382, 487 and 667 bp). *C+* Internal positive control

The virtual gels for each primer set and the duplex of the two primer sets are presented in Fig. 2, showing unique species-and hybrid-specific amplicon profiles.

### T-ARMS-PCR assay testing and optimization

The ITS and 18S primer sets were tested using different ratios of DNA of the two *Schistosoma* species that the primer sets were aimed at distinguishing: *S. bovis* or *S. curassoni* from *S. haematobium*, and *S. bovis* from *S. curassoni* or *S. haematobium*, with DNA ratios of 100/0, 95/5, 90/10, 75/25, 50/50, 25/75, 10/90 and 0/100 for each species combination. The DNA ratios were to simulate differences in allele frequencies in the 18S and ITS targets when the two *Schistosoma* species under investigation have hybridized. This strategy for the DNA combination testing was designed to allow testing of species and species combinations through a process of elimination; for example, for unknown samples the ITS T-ARMS-PCR profile will show that the sample is *S. haematobium* and/or *S. bovis* or *S. curassoni* and then the 18S T-ARMS-PCR profile will allow for discrimination between *S. bovis* and *S. curassoni* and vice versa.

For each primer set, a range of annealing temperatures, primer concentrations and number of PCR cycles were tested. The ability of each primer set to discriminate the different 18S and ITS genotypes was first tested separately on all species combinations and hybrids. The PCR parameters that gave the best results were chosen for the duplex T-ARMS-PCR, which included both the ITS and 18S primer sets (i.e. all 8 primers). The T-ARMS-PCRs, simplex and duplex, were performed in a total reaction volume of 25 µl containing 1× Green Buffer, 1.5 mM of MgCl_2_, 0.2 mM of each dNTP, 0.2 µM of the outer primers, 0.4 µM of inner primers, 2 U of GoTaq® G2 Hot Start (Promega, Madison, WI, USA) and 2 µl of the DNA template(s); PCR grade water was added as needed to reach the reaction volume. Amplification was carried out in a Biometra TOne 96 thermocycler (Biometra GmbH, Göttingen, Germany) using the following cycling programme: initial denaturation at 94 °C for 1 min, followed by 28 cycles consisting of denaturation at 94 °C for 1 min, annealing at 62 °C for 45 s, extension at 72 °C for 40 s, with a final extension at 72 °C for 6 min. Samples (5 μl) of the PCR products were electrophoresed in a 1.8% agarose gel with 1.25 µl of MIDORI Green Advance stain (Nippon Genetics Europe GmbH, Düren, Germany) for each 50 ml of agarose gel, for 40 min at 135 V; amplicons were subsequently visualized using UV light transilluminator (Thermo Fischer Scientific). Genomic DNA (gDNA) from all 169 specimens listed in Table 1 were analysed using the T-ARMS-PCR duplex assay. The genotypes of 148 of the 160 field-collected *Schistosoma* samples were also confirmed by Sanger sequencing of the ITS and 18S amplicons that were generated using our T-ARMS-PCR outer primers (see Table 3).

## Results

### Sensitivity of the T-ARMS-PCR assays

**Fig. 3:**
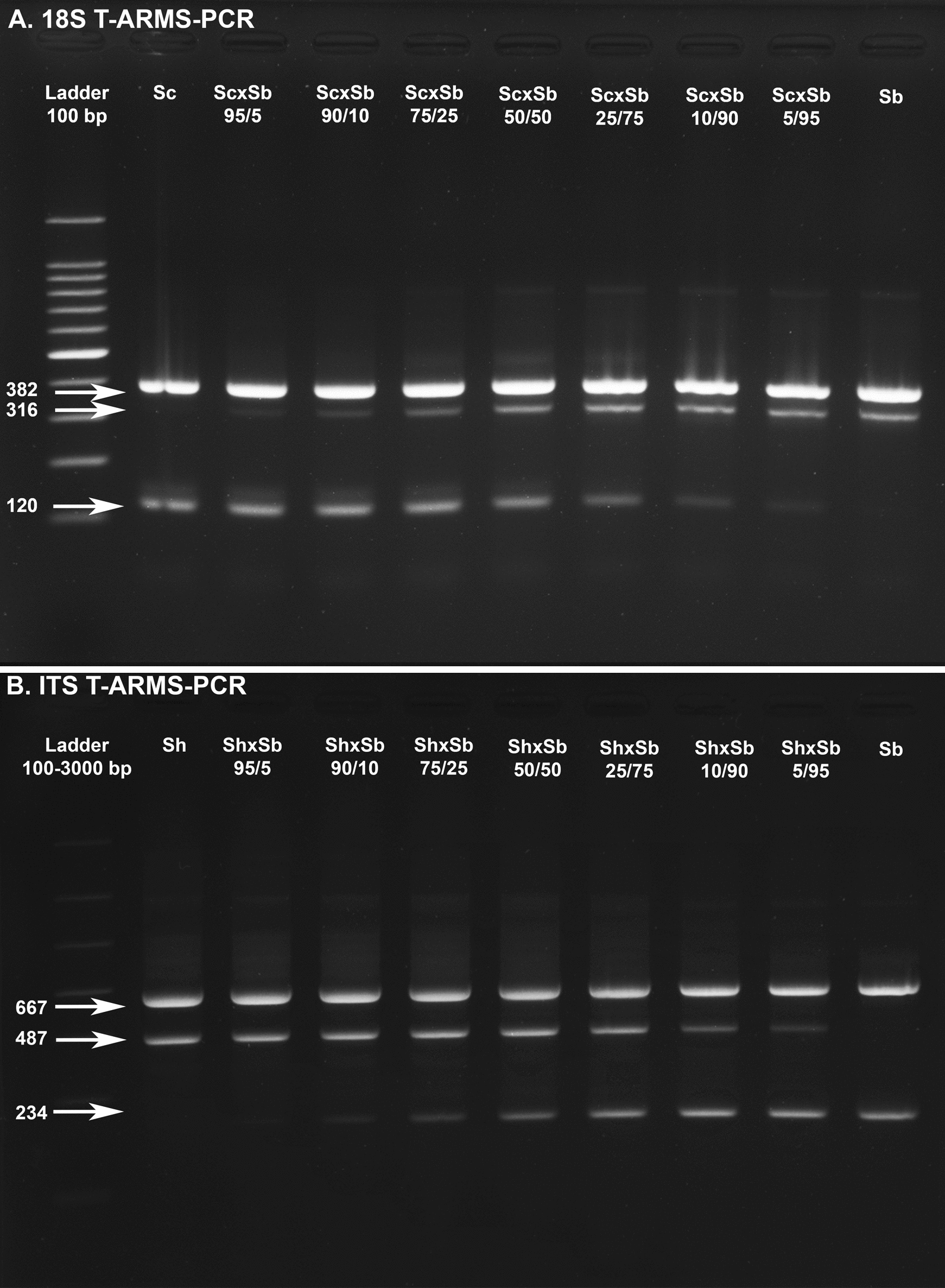
Agarose gel electrophoresis for each T-ARMS-PCR. **a** 18S T-ARMS–PCR assay for different *S. curassoni/S. haematobium* DNA ratios, **b** ITS T-ARMS-PCR assay for different *S. bovis/S. haematobium* DNA ratios. The DNA ratios were 100/0, 95/5, 90/10, 75/25, 50/50, 25/75, 10/90 and 0/100 for each species combination. Each 5% of DNA in the ratio = 0.05 ng of DNA

For the 18S-T-ARMS-PCR (Fig. 3a) the amplicon lengths were 382 bp for the *Schistosoma* internal positive control, 316 bp for *S. bovis* and 120 bp for *S. curassoni* and/or *S. haematobium*. For the ITS-T-ARMS-PCR assay (Fig. 3b), the amplicon lengths were 667 bp for the *Schistosoma* internal positive control, 487 bp for *S. haematobium* and 234 bp for either *S. bovis* and/or* S. curassoni*. For both the 18S and ITS T-ARMS-PCRs, all mixed samples (simulated hybrids) for all DNA ratios presented the amplicons related to both the *Schistosoma* species being tested. As expected, a slight decrease in amplicon intensity was observed when the concentration of gDNA for each species in the ratio was, for example, 95/5 and 90/10). The 18S-T-ARMS-PCR for *S. haematobium* and the ITS-T-ARMS-PCR for *S. curassoni* presented the identical profiles to that of *S. bovis* and *S. haematobium*, respectively (Fig. 3a, b).

### Duplex T-ARMS-PCR assay

**Fig. 4:**
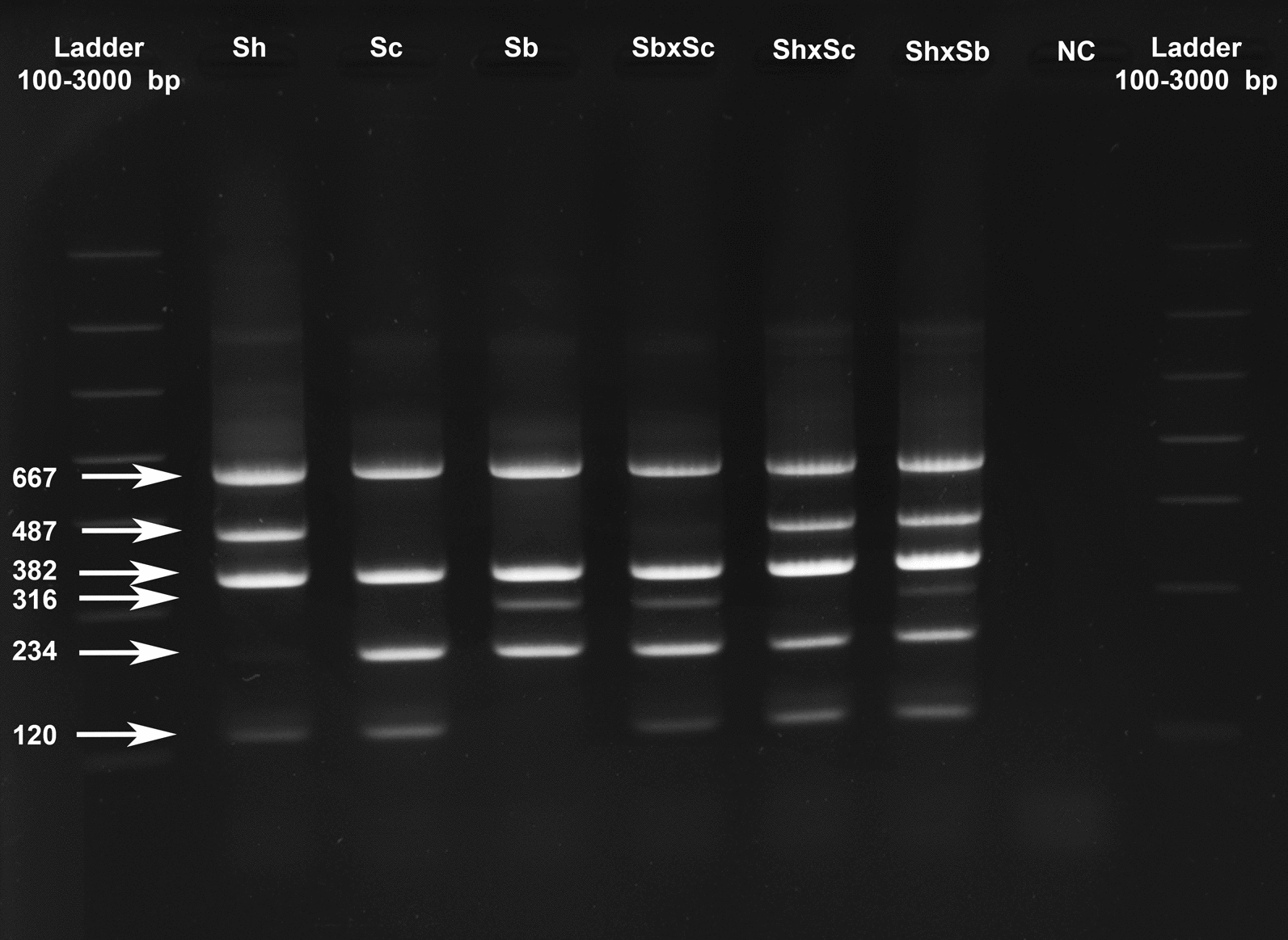
Agarose gel showing the amplicons from the duplex T-ARMS-PCR.* Sh* *S. haematobium* adult from laboratory in Egypt,* Sc* *S. curassoni* field-collected adult from Mali,* Sb* *S. bovis* field-collected adult from Mali,* Sb×Sc* combination shown based on 1 ng of DNA of each species,* Sh×Sc* field-collected hybrid miracidium from Mali,* Sh×Sbl* F1 hybrid from laboratory in Egypt/Spain,* NC* negative control

The duplex T-ARMS-PCR assay results for each species or combination of species are shown in Fig. 4. The amplicon profiles are as expected, as shown in the virtual gel (Fig. 2c). After checking for the presence of the control bands (382-bp 18S band and 667-bp ITS band), the pure species amplicons are 120 and 487 bp, 120 and 234 bp and 234 and 316 bp for *S. haematobium*,* S. curassoni* and *S. bovis,* respectively. The hybrid profiles are 120, 234 and 316 bp, 120, 234 and 487 bp and 120, 234, 316 and 487 bp for *S. bovis* ×* S. curassoni*,* S. haematobium* ×* S. curassoni* and *S. haematobium *×* S. bovis*, respectively. The duplex T-ARMS-PCR assay was validated on the 169 samples detailed in Table 1. Of these 148 of the 160 field-collected *Schistosoma* samples, regardless of the life-cycle stage (miracidium or adult), or the crosses, were validated by sequencing the ITS and the 18S markers and gave 100% concordance with the genotype obtained with the T-ARMS-PCR assay.

## Discussion

Hybridization between parasites is an emerging public health concern, and methods that enable low-cost, rapid and reliable hybrid determination are urgently needed. In the present study, we report the development of a rapid, reliable, and simple duplex T-ARMS-PCR assay that enables discrimination of *S. haematobium*,* S. bovis*,* S. curassoni* and their hybrids. The T-ARMS-PCR genetic profiling of 148 *Schistosoma* samples from Spain, Egypt, Mali, Senegal and Ivory Coast achieved 100% specificity and sensitivity, which included species and hybrid identification. The T-ARMS-PCR assay also proved efficient when the DNA ratio was strongly biased (95/5) towards one species or the other, suggesting that even when there is a strong allelic bias, the T-ARMS-PCR should be able to detect multiple hybrid generations and backcrosses. The T-ARMS-PCR is one of the most frequently used SNP genotyping methods as it requires only standard molecular biology equipment and no additional time after amplification [30, 50]. The T-ARMS-PCR that we have developed genotypes a single SNP on each of the ITS and 18S DNA regions with eight primers in a single PCR reaction. These two SNPs were targeted for the development of the T-ARMS-PCR assay as they met the necessary conditions. Most other SNPs on the 18S and ITS fragments were too close to the end of the sequences and would not provide fragments of sufficient size for identification. Also, other SNPs cannot be used to discriminate the three *Schistosoma* species being targeted, or the SNPs were weak mismatches (G/A or T/C for strong specificity) [30]. Multiplexing the two T-ARMS-PCRs (18S + ITS) is more complex than optimizing single reactions; however, it allows full genotyping of our three *Schistosoma* species and their hybrids in a single reaction. Very few published studies have developed multiplexed T-ARMS-PCR assays. Three multiplexes genotyping two SNPs each were developed for the detection of six mutations associated with cancer diseases [51]. Lajin et al. [52] genotyped three and then four different SNPs involved in human metabolic pathways by developing triplex and quadruplex T-ARMS-PCR [52, 53]. Optimal primer melting temperatures are essential for the successful allele-specific amplification, and each additional SNP added to an assay affects the temperature range needed, which can be incorporated into the PCR cycle; therefore, full optimization of the PCR parameters is needed. Moreover, the interpretation of the results based on amplicon sizes obtained by gel electrophoresis is critical to the success of the assay; thus, it is important to not multiply fragment numbers and sizes.

For this reason, multiplexing more than two SNPs in a single reaction can be difficult. One possibility to overcome this limitation is to use a chimeric primer-based temperature switch PCR (TSP) strategy coupled with capillary electrophoresis for amplicon separation and the identification of the amplicons. Zhang and Liu used this approach to genotype six SNPs in a single multiplex T-ARMS-PCR reaction, with amplicons differing in size by as little as 10 bp being able to be visualized [54]. Capillary electrophoresis was also coupled with a double quadruplex T-ARMS-PCR to genotype eight SNPs in two separate reactions. The T-ARMS-PCR developed in the present study could be advanced to genotype more inter-species *Schistosoma* SNPs as needed using these advanced methods [55]. The use of capillary electrophoresis can be extended to pooling other markers, such as microsatellites or sex-linked markers. A single PCR or pooling of PCR products (poolplexing) would allow genotyping in a single run using a panel of microsatellite markers and enable checking of hybrid status and determination of parasite sex. The addition of several markers determined in a single reaction saves considerable time and is of long-standing interest for the study of genetic populations [8, 11]. Moreover, the addition of genotyping markers is of particular importance in the case of highly introgressed hybrids [11, 35]. However, the use of capillary electrophoresis would be limited by its cost in low-income countries. The T-ARMS-PCR developed in the present study enables identification of the nuclear profiles of *S. haematobium*, *S. bovis*, *S. curassoni* and their hybrids (i.e. heterozygous SNPs). However, it only provides half the genetic profile needed for complete identification as mitochondrial genotyping is also needed [45]; for example, the* cox*1 RD-PCR developed by Webster et al. [45] cannot differentiate *S. bovis* from *S. curassoni*, although it could be useful to develop a T-ARMS-PCR to differentiate these two species at the mitochondrial level. However, mitochondrial molecular markers, such as the *cox*1, are more polymorphic than the nuclear markers ITS and 18S and so the development of a T-ARMS-PCR would be very difficult with the former, with specificity being a potential issue. Design would rely on a large database of mitochondrial genotypes for each species to clearly identify discriminant inter-species SNPs and intra-species regions of homology.

## Conclusions

In this study we provided a rapid, reliable and cost-effective method to discriminate three species of *Schistosoma* (*S. haematobium*, *S. bovis* and *S. curassoni*) and their hybrid forms. The T-ARMS-PCR duplex (18S + ITS) assay which genotypes two nuclear markers in a single reaction allows considerable time and accuracy savings compared to methods currently used for the same purpose. We believe that T-ARMS-PCR assay can significantly improve the study of population genetic studies and evolution of introgression events. In the future, it would be interesting to develop this method on mitochondrial markers to gain even more precision in the genotyping of *Schistosoma* parasites.

## Data Availability

All data generated or analysed during this study are included in this published article.

## Abbreviations

*cox*1: Cytochrome oxidase subunit 1 gene
HRM: High-resolution melting
ITS: Internal transcribed spacer
LAMP: Loop-mediated isothermal amplification
NGS: Next-generation sequencing
PCR–RFLP: Restriction fragment length polymorphism analysis of PCR-amplified fragments
RD-PCR: Rapid diagnostic multiplex PCR
SNP: Single nucleotide polymorphisms
T-ARMS-PCR: Tetra-amplification refractory mutation system PCR
